# Reliability of visual assessment of neonatal jaundice among neonates of black descent: a cross-sectional study from Tanzania

**DOI:** 10.1186/s12887-021-02859-x

**Published:** 2021-09-03

**Authors:** Ikunda Dionis, Omary Chillo, George M. Bwire, Calvin Ulomi, Manase Kilonzi, Emmanuel Balandya

**Affiliations:** 1 Department of Physiology, School of medicine, Muhimbili University of Health and Allied Sciences, P O Box 65001, Dar es Salaam, Tanzania; 2 Department of Physiology and Biochemistry, College of Health Science, (CHS), The University of Dodoma, P.O. Box 259, Dodoma, Tanzania; 3 Department of Pharmaceutical microbiology, School of Pharmacy, Muhimbili University of Health and Allied Sciences, P O Box 65013, Dar es Salaam, Tanzania; 4 Department of Clinical Pharmacy and Pharmacology, School of Pharmacy, Muhimbili University of Health and Allied Sciences, P O Box 65013, Dar es Salaam, Tanzania

**Keywords:** Neonatal jaundice, Black descent, Kramer, Diagnostic accuracy, Sensitivity, Specificity

## Abstract

**Background:**

Jaundice is common among neonates and if untreated can lead to kernicterus. Diagnosing neonatal jaundice (NJ) using Kramer’s method (visual assessment) is considered user-friendly in resource-limited areas. However, there are conflicting findings on reliability of the Kramer’s method in the diagnosis of NJ, particularly of black descent. Therefore, study aimed to determine the accuracy of Kramer’s method in comparison to the total serum bilirubin (TSB) test in the diagnosis of NJ among neonates of black descent in Tanzania.

**Methods:**

A cross-sectional study was conducted between June and July 2020 at Muhimbili National Hospital (MNH) in Dar es Salaam Tanzania. A total of 315 neonates were recruited consecutively. In each neonate, jaundice was assessed using Kramer’s method and TSB test. NJ A total of 315 neonates were recruited i. A 2 X 2 table was created for the determination of sensitivity, specificity, positive predictive value (PPV), negative predictive value (NPV), positive and negative likelihood ratios (+LR/−LR), and diagnostic accuracy (effectiveness) of Kramer’s method. Cohen kappa (κ) was used to analyze the agreement between Kramer’s method and TSB. Association between independent variables and presence of jaundice were assessed using the chi-square test and the *p* < 0.05 was considered to be statistically significant.

**Results:**

The prevalence of NJ was 49.8% by Kramer’s method and 63.5% by TSB. The Sensitivity, Specificity, PPV, and NPV of Kramer’s method were 70.5, 86.1, 89.8, and 62.6%, respectively. The +LR and –LR were 5.07 and 0.34, respectively. The diagnostic accuracy of Kramer’s method was 76.1%. There was a moderate agreement between Kramer’s method and TSB results (κ = 0.524, P<0.001). No significant relationship was observed between the independent variables and the presence of NJ.

**Conclusion:**

Kramer has a good positive predictive value. However, due to low sensitivity and NPV one cannot say that overall predictive ability is good. Also, clinical assessment by Kramer’s method should not be used for screening of NJ. Further studies are needed to investigate the utility of other non-invasive techniques in detecting NJ among neonates of black descent.

## Background

Neonatal jaundice (NJ) affects one in two neonates worldwide [1] and the problem is much bigger in sub-Saharan Africa and South Asia. About 60% of the term and 80% of the preterm babies suffer jaundice during the first 7 days of life and is the major reasons why neonates attend emergency department [2, 3]. Neonates with jaundice are prone to bilirubin encephalopathy and kernicterus which contribute to neonatal morbidity and mortality [4].

Early detection/diagnosis and appropriate management of NJ are very crucial in order to prevent its complications. Visual assessment (Kramer’s rule), transcutaneous bilirubinometry (TcB), and TSB are the approaches used for the detection of NJ [5].

Kramer’s method is user friendly because it is non-invasive, does not require any machine and so can be used effectively in resource limited settings. The technique was introduced by Kramer, and showed a positive correlation between progressions of skin discoloration and serum bilirubin levels [5, 6] due to skin thickness differences. According to Kramer, the assessment should begin from the head towards the feet which portray the five zones of the cephalo-caudal progression of jaundice. It is important to examine all zones because studies reported that bilirubin concentration differ significantly between each dermal zone.

Controversial findings on reliability of this technique for detecting NJ have been reported worldwide. Studies conducted in Pakistan (2010) and Indonesia (2017) reported that Kramer’s method is reliable in detection of NJ and darker skin color is not a matter of concern [7, 8]. However, a study conducted in United State of America (USA) reported that Kramer’s method alone is not reliable in detecting NJ especially in darkly pigmented neonates [9]. Besides, study conducted in South Africa reported that Kramer’s method was able to pick only 17% out of the 52% neonates with hyperbilirubinemia [1].

Despite those conflicting results, Kramer’s method is the backbone in diagnosis of NJ in most of the low-level health facilities in low and middle income countries like Tanzania. Therefore, this study aimed to determine the sensitivity, specificity and accuracy of Kramer’s method in detecting NJ in comparison with TSB test in Tanzania setting.

## Methods

### Study design, period and setting

A hospital-based cross-sectional study aimed at assessing the reliability of the Kramer’s method in detecting NJ was conducted between June and July 2020 at Muhimbili National Hospital (MNH), Tanzania. MNH is a national hospital located in Dar es Salaam center, receiving patients from different parts of the city and regions of Tanzania. The neonatal unit at MNH is one the biggest in the country, serving up to 500 neonates per month. The unit has adequate numbers of skilled pediatricians, medical doctors and nurses. In addition, the MNH has a well-equipped central laboratory with capacity of performing numerous tests, including total serum bilirubin.

### Study population and eligibility criteria

Black descent neonates aged less than 28 days who were admitted at MNH and whose mothers/guardians provided written consent to participate in the study were enrolled. Neonates with rashes and those who were severely sick (in life support, in oxygen masks) were excluded because assessment of NJ using Kramer’s method cause physical discomfort to the babies.

### Sample size, sample size calculation and sampling technique

A total of 315 neonates were recruited into this study. The formula for ‘diagnostic sample size calculation’ was used to calculate the sample size [10] (i.e. *n* = Z^2^ x Specificity x (1-specificity) / e^2^ x (1-prevalence)). Specificity of Kramer’s method of 89% from a study conducted in Indonesia [8], prevalence of NJ of 50% in Tanzania (unpublished article), precision of 5 and 95% (z = 1.96) confidence interval were used to obtain 283.5. Considering 10% non-respondent rate, a total of 315 neonates were obtained. Participants were recruited consecutively.

### Data collection procedure

Data were collected using case report form (CRF) comprising of 3 sections; demographic information (age, gender, birthweight, gestational age, and delivery mode), Kramer scores and TSB results. The CRF was developed following literature review and experts’ consultation. Each neonate was subjected to visual assessment using Kramer’s method and TSB test. Neonatal jaundice was defined by yellowish discoloration of the skin by visual assessment or TSB level of ≥ 85 μmol/l by TSB [11].

### Visual assessment using Kramer’s rule

Two medical doctors and 1 pediatrician with experience in neonatal assessment performed the visual assessment on routine ward round. For each neonate, visual assessment using Kramer’s method was performed by the 2 doctors separately and discrepancies were resolved by the pediatrician. Visual assessment of the 5 dermal zones were scored as no jaundice =0, jaundiced at level of head and neck = 1, jaundiced at upper trunk (above umbilicus) =2, jaundiced at lower trunk and thighs (below umbilicus) =3, jaundiced at arms and lower legs =4, jaundiced palms and soles = 5 [12]. The discrepancy of one value in Kramer score was acceptable. Discrepancies of more than one value in Kramer score were resolved by consulting a pediatrician. Kramer score recorded for each neonate was the average score of the two examiners. During assessment, the neonate was fully undressed, and assessment was conducted under blue fluorescent light. Besides, by using the thumb, the skin of neonate was blanched to observe the underlying skin color from the head to toes following standard procedure described by Devi et al. [6].

### Laboratory measurements

About 2–2.5 mls of blood sample was withdrawn from each neonate’s femoral vein using 2.5 cc syringe into a green topped heparinized vacutainer tube. The collected sample was transported to the laboratory for processing within 2 h using a cooler box containing ice packs. In the laboratory, the sample was centrifuged using a centrifuging machine to obtain serum. The obtained serum was analyzed for bilirubin concentration using the spectrophotometric chemistry analyzer (Architect chemistry analyzer, USA). Bilirubin determination was based on the run of bilirubin with diazotized sulfanilic acid. Azobilirubin concentration was measured at an absorbance of 500-600 nm which is proportionate to bilirubin concentration.

### Data analysis

Data was entered and analyzed by SPSS version 23 software. Frequencies and percentages were used to summarize categorical variables and continuous variables were summarized by using median (interquartile ranges (IQR)). For Kramer’s method, those who scored 0 were grouped as no jaundice while 1–5 were considered to have jaundice. TSB test of ≥85 μmol/l were considered to have jaundice and below that as no jaundice. Sensitivity, specificity, predictive values and diagnostic effectiveness (accuracy) were obtained by contingency Tables (2 × 2 table for diagnostic test) [13]. Diagnostic accuracy results were defined as very weak (> 50–60%), weak (> 60–70%), moderate (> 70–80%), good (> 80–90%), or very good (> 90–100%). Positive likelihood ratio (+LR) >10 and negative likelihood ratio (−LR) < 0.1 were considered to be a good test [14]. Cohen kappa statistic (κ) was used to determine agreement of Kramer’s method and TSB results, with (κ) of 0.21–0.40, 0.41–0.60, 0.61–0.80 and 0.81–1.00 being fair, moderate, good and very good agreement, respectively [13]. Association of independent variables and NJ was done using the chi-square test (X^2^ test) and *p* < 0.05 was considered to be statistically significant.

## Results

**Fig. 1:**
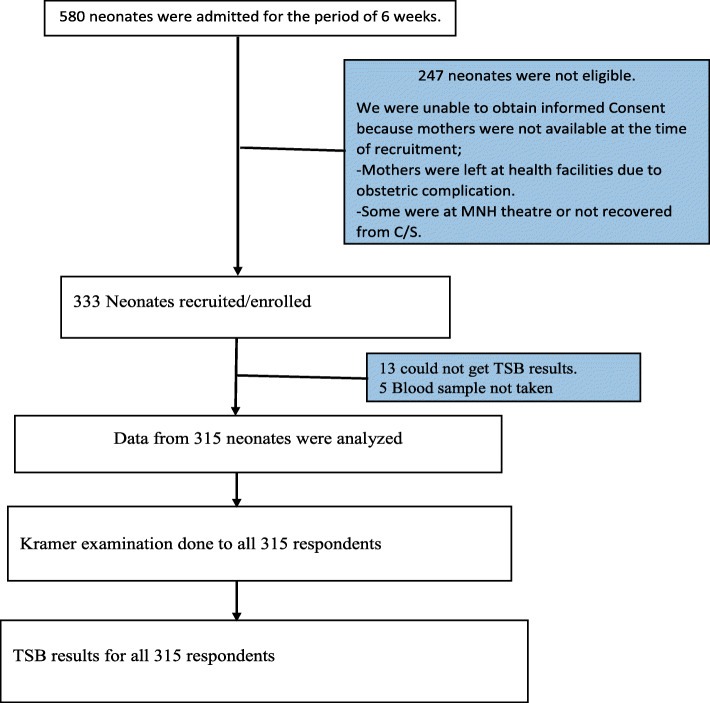
Schematic of participants’ enrolment

580 neonates were admitted at the MNH neonatal unit during the study period. 247 were not eligible because written consent was not obtained as mothers were left at health facilities where they gave birth due to obstetric complications/management and 333 were enrolled. Out of 333, 18 were excluded because blood samples were not taken [13] and TSB test results were misplaced [5]. Therefore, a total of 315 were subjected to analysis Fig. 1.

### Socio-demographic characteristics of the participants

Of 315 respondents with complete data, 182 (57.8%) were males with the median (IQR) age of 4 [2–8] days. More than half (58.4%) of the neonates were delivered by spontaneous vaginal delivery and half of the neonates 158 (50.2%) were born at preterm with median (IQR) birth weight of 2500 (1700–3000) grams Table 1.

**Table 1:** Social-demographic characteristics of the participants (*n* = 315)

Variables	Median(IQR)	n (%)
Median age (IQR) years	4(2-8)	
Age group(days)		
1 to 7		233(74.0)
> 7		82(26.0)
Gender		
Male		182(57.8)
Female		133(42.2)
Mode of delivery		
Spontaneous vaginal delivery (SVD)		184(58.4)
Cesarean section		131(41.6)
Median birth weight (IR)	2500(1700-3000)	
Birth weight group		
Normal birth weight (NBWT)		163(51.4)
Low birth weight (LBWT)		152(48.6)
Gestation age(GA) group		
Term		157(49.8)
Preterm		158(50.2)
Visual assessment (Kramer)	Median TSB(IQR)	n (%)
Zone 0	65.2(40.8-106)	158(50.2)
Zone 1	110(73.4-211.9)	17(10.8)
Zone 2	131(91.0-200.1)	27(17.2)
Zone 3	182(129.8-209.5)	43(27.3)
Zone 4	206.6(137.1-302)	47(29.9)
Zone 5	264.8(154.6-314.7)	23(14.6)

*IQR* Interquartile range, *SVD* Spontaneous Vaginal delivery, *NBWT* Normal birth weight ≥ 2500 grams, *LBWT* Low birth weight ≤2499 grams, *GA-Gestational age* Term-born from GA of37 weeks and above, Preterm- born below GA of 37 weeks

### Magnitude of NJ by Kramer’s method, TSB and associated factors

The prevalence of NJ was 63.5% by TSB and 49.8% by Kramer’s method. The median (IQR) TSB for neonates who were clinically not jaundiced was 65(40–106) μmol/l and 178 (121–238) μmol/l for neonates who were clinically jaundiced. Following chi-square test, all variables including age (*p* = 0.46), gender (*p* = 0.77), delivery mode (*p* = 0.13), birthweight (*p* = 0.19) and gestational age (0.78) were not significantly associated with the occurrence of NJ.

### Diagnostic accuracy of the Kramer’s method

Kramer’s method had a Sensitivity-70.5%, Specificity-86.1%, PPV-89.8%, and NPV-62.6% in detecting NJ. It had 5.07 LR of detecting jaundice and 0.34 LR of not detecting jaundice among neonates with a diagnostic accuracy of 0.761. Kramer’s method had moderate agreement with TSB test (κ = 52.4, *P* < 0.01) Table 2.

**Table 2:** Diagnostic accuracy of the Kramer’ method

TSB outcome	Kramer outcome		Total	*p*. value
	Positive	Negative		
Jaundiced	141(70.5)	59(29.5)	200	0.001
Not jaundiced	16(13.91)	99(86.1)	115	
**Measures of diagnostic performance**		**Value**		
Sensitivity (95% CI) (%)		70.5(63.84 to 76.38)		
Specificity (95% CI) (%)		86.1(78.59 to 91.25)		
Positive predictive value (95% CI) (%)		89.8(84.09 to 93.06)		
Negative predictive value 95% CI) (%)		62.6(54.89 to 69.82)		
Diagnostic accuracy (95% CI)		0.761		
Likelihood ratio of a positive test		5.07		
Likelihood ratio of a negative test		0.34		
Cohen’s kappa, *p* value		52.4, < 0001		

### Diagnostic performance of Kramer’s method based on term, preterm, and birthweight

For neonates born at term, the Kramer test had 75.3% sensitivity, 82.8% specificity, 86.4% PPV, 69.7% NPV and 56.4% Cohen kappa in comparison to 66.4% sensitivity, 82.8% specificity, 93.0% PPV, 56.0NPV and 48.8% Cohen kappa for preterm neonates, *P* < 0.001 Table 3.

**Table 3:** Diagnostic performance of visual assessment based on Term, Preterm, and birthweight

Kramer test	ALL(***n***=315)	Neonatal sub-group			
		Term(***n***=157)	Preterm(***n***=158)	NBWT(***n***=163)	LBWT(***n***=152)
Sensitivity	70.5	75.3	66.4	80.0	61.9
Specificity	86.1	82.8	82.8	83.6	89.4
PPV	89.8	86.4	93.0	87.3	92.8
NPV	62.9	69.7	56.0	75.0	51.8
Cohen Kappa (κ)	52.4	56.4	48.8	62.8	42.5

*PPV* Positive predictive value, *NPV* Negative predictive value, *NBWT* Normal birth weight ≥ 2500 grams, *LBWT* Low birth weight ≤2499 grams, Term- Delivery at GA of 37 and above weeks, Preterm- Delivered below GA of 37 weeks.

## Discussion

The study aimed at determining the reliability of Kramer’s method for assessment of NJ among neonates of black descent. Our study found the Kramer’s method to have moderate diagnostic accuracy (76%), moderate sensitivity (70.5%), and good specificity (86.1%). Besides, Kramer’s method was found to have good ability of predicting presence of NJ (89.8%).

Our findings are consistent with the study conducted in Indonesia, in which sensitivity and specificity of Kramer’s method reported to be 76.92 and 89.47% respectively. This study was conducted among neonates of black descent and the diagnostic accuracy found to be moderate which is in line with findings from various studies that reported the diagnostic accuracy of Kramer’s method to be poor for neonates of black ethnicity compared to other groups [15]. Hence, our study emphasizes that Kramer’s method may be used as a predictor (PPV =89.8%) of NJ rather than confirmatory test for the presence of NJ. In contrast to our findings, the Indonesian study reported good (86.27%) diagnostic accuracy of Kramer’s method [8]. The observed discrepancy could be due to differences in sample size and characteristics of study participants since our study involved only neonates of black descent while the study in Indonesia included neonates of black and white descent. In addition, our findings are similar to what was reported in Switzerland in which the diagnostic accuracy of Kramer’s method was found to be moderate (73%) [15].

Our study also demonstrated slightly increases in sensitivity of Kramer’s method when detecting jaundice among term (75.3%) and normal birthweight (80%) neonates and increase in specificity among neonates with low birthweight (89. 4%). These findings are comparable to what was reported in Denmark and Switzerland where sex, birthweight and gestational age were demonstrated to be the determinants of the diagnostic accuracy of the visual assessment technique [15, 16]. These findings suggest the consideration of gestational age and birthweights when using Kramer’s method in predicting NJ.

TcB, a non-invasive technique, can be used to detect NJ at an early stage. In comparison to TSB, this technique is able to detect bilirubin concentration easily, does not cause heel-puncture pain or irritation to the child, and is simple to calibrate. TcB, however, is unable to detect accurate bilirubin concentrations after 10 mg/dl, necessitating TSB confirmation. Furthermore, since the TcB machine costs thousands of dollars, it is not readily available in resource-limited settings like Tanzania. Similarly, since hemoglobin, melanin, skin types, and age all affect readings, most non-invasive methods cannot be used to diagnose severe disease [17–19].

Lastly this study showed that the occurrence of NJ was independent of age, gender, delivery mode, birthweight and gestational age. These findings are comparable to a study done by Aprillia et al. (2017) which reported that gender, skin color and gestational age were not the determinants for the occurance of NJ. However, our findings are inconsistent with a review conducted in USA (2011) which reported that physiological jaundice was expected to peak between 3 and 5 days of life [20]. Additionally, a study conducted in India showed that hyperbilirubinemia was much more common in female than male neonates because albumin concentration differs between gender [16]. The observed difference might be due to differences in study population as the review in USA only included neonates of less than 5 days of life and study in India recruited only neonates with low birthweight).

The fact that this study was conducted at the tertiary referral hospital implies that the prevalence of NJ might have been overestimated. Also, Kramer method could be affected by intra- observer variability which was tacked by reconciling individual observations to the level of one-unit difference in scores, and computing Cohen kappa statistics for reliability during data analysis.

## Conclusions

Kramer has a good positive predictive value. However, due to low sensitivity and NPV one cannot say that overall predictive ability is good. Also clinical assessment by Kramer’s method should not be used for screening of NNJ. Further studies are needed to investigate the utility of other non-invasive techniques in detecting NJ among neonates of black descent.

## Data Availability

The datasets analyzed during this study are available from the corresponding author on reasonable request.

## Abbreviations

CI: Confidence interval
CRF: Case report form
CS: Cesarean section
GA: Gestational age
IQR: Interquartile range
LBWT: Low birth weight
LR: Likelihood ratio
MNH: Muhimbili national hospital
MUHAS: Muhimbili university of health and allied sciences
NBWT: Normal birth weight
NJ: Neonatal Jaundice
NPV: Negative predictive value
PPV: Positive predictive value
SPSS: Statistical product and service solution
SVD: Spontaneous vaginal delivery
TcB: Transcutaneous bilirubinometry
TSB: Total serum bilirubin
USA: United states of America
